# Socio-Economic Impacts of COVID-19 on Working Mothers in France

**DOI:** 10.3389/fsoc.2021.732580

**Published:** 2021-12-17

**Authors:** Anne Lambert, Violaine Girard, Elie Guéraut

**Affiliations:** ^1^ Institut National d’études démographiques, Paris, France; ^2^ Université de Rouen-Normandie, Mont-Saint-Aignan, France; ^3^ Université de Clermont-Auvergne, Clermont-Ferrand, France

**Keywords:** COVID-19 and gender, family well-being, housing and living conditions, lockdown, working mothers in France

## Abstract

Beyond its devastating consequences for public health, the COVID-19 pandemic had a major impact on gender inequalities, labour markets and families. Compared to many European countries, the French approach to lockdown was among the more stringent, although the measures taken by the French government to support employment, to some extent, mitigated the worst effects of the crisis on families. This article analyses the implications of COVID lockdown restrictions on gender equality and well-being for couples with children in France. The study adopted a multidimensional approach to gender inequalities associated with paid work and various dimensions of living conditions, involving gender-differentiated access to personal work spaces in the home, personal leisure time outside the home, and local support networks during the first phase of lockdown (March−June 2020). Drawing on data from the COCONEL survey, carried out by the Institut national d’études démographiques on a quota sample of the French adult population in April/May 2020, the authors controlled for variables including socio-economic status, age, family structure and place of residence. The survey data were complemented by a longitudinal set of in-depth interviews enabling the research team to capture the differential effects of the pandemic within couples. The main findings indicate that, despite the frequency of dual-employment arrangements for heterosexual couple households with dependent children, French mothers were nevertheless more likely to reduce their working time and/or withdraw from the labour market. Within the households surveyed, mothers were less likely than fathers to leave the home during the day, particularly for personal leisure activities. The presence of children in households increased gender inequality in both employment and living conditions across all socio-economic categories. In conclusion, the authors consider whether the pandemic might have a long-term impact on gender norms and inequalities within families, and how the findings about changes in gender inequalities could be used to inform public policy development.

## Introduction

The COVID-19 pandemic had a major impact across the world, not only on health, particularly for older people and those with underlying health conditions, but also on social life and labour markets, where it caused mass layoffs, job dislocation, and income loss. The effects of the crisis were highly uneven both between and within countries, depending on working arrangements and workers’ characteristics (education, socio-occupational category, gender), as well as on the public policies implemented.

France occupies an intermediate position in Europe regarding the socio-economic impacts of the COVID-19 crisis. It was one of the EU member states hardest hit during the first wave of the pandemic and was characterised by the stringency of the measures implemented (Oxford Blavatnik School of Government, 2020). From 6 March 2020, childcare services, schools and universities were closed, even though special arrangements were organised for children with parents who were key workers. From 17 March to 11 May, national lockdown measures were applied, and a state of health emergency was established on 24 March. Enterprises closed except for essential services. The French economy contracted substantially in the second quarter of 2020: GDP fell by 13.8%, which was more than the eurozone average. In the first half of the year, 715,000 jobs were lost, most of them in the last 2 weeks of March (Barhoumi et al., 2020). As in other European countries, the government implemented specific measures to support the economy and employees, and to avoid mass layoffs. From 24 March, compensation schemes were provided for employees forced to stay home and unable to telework, either because their enterprises had closed or because they had to care for children under the age of 16 (Legifrance, 2020). This article focuses on the impact that these lockdown measures had on working mothers and on their consequences for gender inequality in France.

### Mechanisms Exacerbating Gender Disparities

Three types of mechanisms have been highlighted in studies to explain the rise in gender inequalities during the COVID-19 crisis and the diverse effects of the pandemic across and within countries. The first is linked to the form and intensity of labour market segregation. In Europe, women are over-represented in the public-facing service sectors (hospitality, tourism, retail, welfare) that were disproportionately affected by closures due to social distancing and lockdown measures, and in cases where they were less able to work from home (Blasko et al., 2020; Fana et al., 2020). Moreover, women, especially those with lower levels of education, are over-represented in non-standard work, including temporary, part-time and agency employment, which are typically poorly paid and are sometimes exempt from direct social security cover. In the United Kingdom and to a lesser extent in Germany (two countries where real-time survey data on employment during COVID19 period are available), the proportion of women in these types of jobs is relatively high, and women were on average more likely to be adversely affected by the crisis. The smaller proportion of women in non-standard jobs in France compared to Germany and the United Kingdom suggests that poorly educated women in France might have been expected to be less adversely affected by the crisis than in these two countries. But some studies have suggested that they were more likely to be affected than more highly educated women (Weinkopf, 2015; Adams-Prassl et al., 2020).

The second mechanism refers to the social norms determining acceptable roles for women in society and the household. Although the dual-breadwinner family model has become dominant in Europe, the share of unpaid work within households remains largely unequal (Blasko et al., 2020). In most dual-earners couples, women had long been the lower-earning spouse, largely because more women than men work part-time, and more women take parental leave (Morin, 2014). However, cross-country disparities are observed within Europe. The “full-time dual-earning” model was more widely adopted in France than in Germany or the United Kingdom, where the “one-and-a-half-earner” model long remained dominant with one parent, usually the father, working full-time and the other, often the mother, working part-time (OECD, 2017). In France, two-earners households accounted for 60% of all partnered households in the early decades of the twentieth century. Before the pandemic, 75% of women aged 25–49 with children under 15 were in employment, compared to 84% for those without children (Bentoudja and Razafindranovona, 2020). The relative prevalence of the dual-earner model in France compared to the United Kingdom and Germany suggests that the consequences of COVID-19 on gender inequalities might have been mitigated to a greater extent in France than in other Western European countries.

Third, cross-country gendered disparities depend on the public policies implemented, such as school closures, and financial support for workers with children. Employees in Germany, which has a well-established short-time work scheme (Kurzarbeit), were, for example, much less likely to be affected by the crisis than in France or the United Kingdom, where furlough measure were widespread (Adams-Prassl et al., 2020). Short-time work compensation and the “family bonus” increased child benefit for vulnerable families in Germany (Cook and Grimshaw, 2020; Müller and Schulten, 2020). No significant difference was reported in job loss between women and men in Germany, although time-use data showed that women took on more childcare than men even when working from home (Adams-Prassl et al., 2020). In the United Kingdom, the Coronavirus Job Retention Scheme, introduced in March 2020, allowed firms to furlough workers for up to 3 months. The scheme replaced 80% of employees’ wages up to a maximum of £2,500 per month. The German Kurzarbeit scheme prevented furloughed workers from undertaking any work for their employer, and childcare needs were not acknowledged in the provisions made.

In France, women and men who were unable to work owing to workplace, school and childcare service closures, or other lockdown measures, could claim employment insurance or social security payments. Short-time work compensation was high—at the rate of 84% of the previous net salary—and extended to non-standard employment. More than a third of those employed prior lockdown were on short-time work during this period (Givord and Silhol, 2020). Moreover, an emergency flat-rate solidarity allowance was paid to low-income households by the Family Allowances Fund (Caisse d’Allocations Familiales). This allowance applied to 1.4 million households (about 5% of French households) and 5 million children. However, despite a generous support policy during the COVID-19 crisis, compensation for school and childcare closures was provided only to one parent, which may have generated trade-offs between parents within couples.

### Diversity in the Impacts of Lockdown on French Households

In spite of the socio-economic measures taken by the French government at national level to support employed workers, the impact of the pandemic on households varied according to age, socio-economic status (income, education, and occupation) and gender (Lambert and Cayouette-Remblière, 2021). In France, one in three women in employment in March 2020 had stopped working in May 2020, compared to one in four men. Another French study, which does not provide information by occupation and education, revealed that women in employment were twice as likely as men to have stopped working to look after children during the first wave of the pandemic, and that they spent on average more time on domestic and parenting tasks than men (Albouy and Legleye, 2020). During lockdown, contacts with older people were banned as well as intergenerational family visits. Consequently, working parents could no longer rely on informal childcare by grandparents. Within couples, women took on a greater share of domestic tasks than their spouse, irrespective of their employment status during lockdown (Pailhé et al., 2020). An analysis of the disparities in material living conditions and well-being during lockdown in France showed that, on average, women suffered a greater loss in income (Lambert et al., 2020).

The research reported in this article explores the impact of lockdown on working-age mothers in two-adults households with the aim of understanding the interactive effects of gender and parenthood. The study contributes to the literature on COVID-19 and gender inequalities in two ways. After explaining why the French case is of interest for an analysis of the interactive relationship between COVID-19 and the experience of working mothers during the pandemic, the research team sought new evidence demonstrating how the pandemic affected gender inequalities in heterosexual families in France. In contrast to much of the previous literature, the project team adopted a multidimensional approach in analysing developments in gender inequalities during the first wave of the COVID-19 crisis, taking account of paid and unpaid working and living arrangements, and social well-being.

The first research question considers whether, despite substantial public aid in France aimed at preventing mass unemployment and the exit of salaried parents from the labour market, working-age women with children were more adversely affected by the crisis and lockdown measures than men in the same situation, regardless of social category. The second question concerns the negative impact of lockdown on the family and social lives of mothers, and consequently on their well-being. It leads onto an analysis of the relationship between housing conditions, private space and activities outside the home, and the sharing of educational and domestic tasks between parents.

## Materials and Methods

The study draws primarily on cross-sectional data collected in the sixth round of a longitudinal online survey (COCONEL, COronavirus et CONfinement: Enquête Longitudinale). This round of the survey was designed and conducted by the Institut national d’études démographiques (INED), focusing on housing and living conditions during the first wave of the pandemic. A sample of 2,003 adults living in metropolitan France were questioned online between 30 April and 3 May 2020, using a quota sampling method covering age, gender, education, occupation, and category of municipality. Data collected included socio-demographic characteristics, household composition, a detailed description of housing conditions, employment characteristics, and perceptions of well-being.

The COCONEL survey has three advantages compared to other national surveys. It contains information about the situation pre- and post-lockdown, meaning that changes in individual situations can be compared over time. Its approach to living conditions during the crisis was not limited to employment and the division of household work, which were the particular focus in the international literature and several ad hoc surveys in France, such as EpiCOv (Bajos et al., 2020). COCONEL collected separate information about the socio-occupational category of each partner in the couples to capture the household’s social status in terms of lower, middle and higher socio-economic groups.

Supplementing the COCONEL survey, the article draws on in-depth interviews and qualitative longitudinal analyses of families in different types of housing arrangements and social class, enabling an analysis of the subjective experience of the crisis and the mechanisms leading to greater inequalities within couples. The interviews focused on the changes that occurred during the crisis in terms of housing, family, work, and day-to-day life. 21 in-depth interviews were carried out in April and May 2020 by the COCONEL study group by telephone or online owing to the physical distancing measures imposed at that time. They were all recorded and fully transcribed. It is important to note that the interviewees had already been followed and interviewed in person as part of earlier qualitative surveys, which meant that their “regular” living conditions were well known and documented.

This mixed methods approach enabled the authors to reconstitute the dynamics of inequalities in the longer timespan of the life course. Furthermore, by focusing on the domestic sphere, they were able to gain a better understanding of family dynamics and a firmer grasp of the trade-offs made by families in confronting the gendered experience of lockdown.

## Findings

The analysis presented in this article shows that the deterioration in employment and working conditions during lockdown was more pronounced for mothers than for fathers, thereby confirming the observations made in some other countries. In addition, it shows that living conditions were more difficult for mothers than for fathers during this period, in particular because they spent less time outside the home during the day than did fathers. Working-age women with children also complained more often than fathers about their housing conditions. Similarly, experiences of teleworking differed by gender, particularly in better-off households where housing conditions were more amenable to home working.

### From Work Place to Living Conditions at Home

The odds ratios from the COCONEL data analysis in Table 1 show that, among people in employment on 1 March 2020, women in couples with children were 1.456 times more likely than men to have stopped working by May 2020 when controlled for age, socio-economic category and residential area. This result suggests that mothers left the workplace more often than fathers to manage the increase in domestic and parenting tasks generated by the health crisis and lockdown measures, thereby further increasing pre-existing inequalities within families (Champagne et al., 2015).

**TABLE 1:** Logit: work stoppage.

Variables	Odds ratios
Men without children vs. men with children	0.723**
Women without children vs. men with children	1.131
Women with children vs. men with children	1.456**
Middle vs. higher socio-economic groups	1.688
Lower vs. higher socio-economic groups	3.074***
18–25 vs. 25–49	4.850***
50–64 vs. 25–49	1.236**
Small and medium-sized towns vs. rural areas	1.122
Cities vs. rural areas	1.147

*p < .05; **p < .01; ***p < .001.

Economically active in employment at 1 March 2020, in a couple, aged under 65 (*n* = 1,077).

Source, 2021 Source: COCONEL survey, April/May 2021.

The additional household work was performed entirely within the home. Housing conditions and the ways in which domestic space is shared appeared to be decisive in the assessment made by mothers and fathers of the effects of lockdown on the well-being of family members. Overall, the women in the sample population lived in smaller dwellings than the men, with an average of 45 square metres of living space compared to 51 square metres for men, factoring in the number of individuals in the household. This disadvantage was aggravated by the health crisis since more women than men were living with dependent children during lockdown: 36.7% of women lived with at least one dependent child during the period, compared to 29.4% of men. In addition, exposure time to poor housing conditions increased owing to restrictions on leaving the home.

The COCONEL survey showed that women in couples with children had more negative perceptions of their housing conditions during lockdown than men in the same situation. Whereas 13% of all female respondents said their home lacked space, compared to 9% of men, the percentage rose to 18% for women in couples with children, compared to 12% of men in the same situation. Among women and men in couples without children, gender differences are almost non-existent. This differing perception of housing conditions can be attributed to the fact that more women than men stopped work or reduced their working hours during lockdown. They took on greater responsibility for daily household tasks and the material aspects of daily life — cleaning, washing up, laundry, preparation of meals — which meant that they were more sensitive to the lack of living space in everyday life.

### Life Outside the Home

This perception gap also stems from differences between women and men in couples with children in terms of life outside the home. Daily outings were stringently regulated in France during the eight-week lockdown in spring 2020. A list of exemptions applied for work, exercise, essential shopping and health visits; a maximum period of one hour within a radius of one kilometre from the home was enforced for physical exercise, and shopping was confined to basic necessities. But these legal restrictions did not prevent major differences between population categories. In the sample of respondents to the COCONEL survey, respectively 40% of men and 53% of women on average said they did not leave their home on the day preceding the interview. The gender gap was largest among couples with children, with 37% of men in couples with children not having left their home the day before the survey, compared to 53.5% of women (see Table 2).

**TABLE 2:** Outings according to sex and family composition (as a %).

	No outings	One outing	Two outings or more
Men in a couple without children	42.2	40.2	17.6
Men in a couple with children	36.7	36.6	26.7
All men in a couple	40.1	38.8	21.1
Women in a couple without children	54.3	35.0	10.6
Women in a couple with children	53.5	27.4	19.1
All women in a couple	53.9	31.3	14.8
Couples without children	47.6	37.9	14.5
Couples with children	46.1	31.5	22.5

*Coverage:* Individuals in a couple (*n* = 1,233).

The origin of these inequalities can be discerned in the reasons given for leaving the home. Men and women with no children were equally likely to leave the home to go to work, but among couples with children, men left the house to go to work more often (+7.5 points) than women. Gender differences were much more pronounced for recreational and sporting activities. The gap in each case was greater among couples with children. The number of men in couples with children engaging in sporting activities outside the home was three times higher than for women in the same situation. Outings were also longer for men than for women (231 and 184 min per day respectively). The regression model presented in Table 3 shows that men were 1.6 times more likely than women to have left the home the day before the survey interview, regardless of the reason, when controlled for job characteristics, family situation, age, and social milieu.

**TABLE 3:** Logit: leaving the home in the day (all reasons).

Variables	Odds ratios (model 1)	Odds ratios (model 2)
Men vs. women	1.597***	1.557***
Single-parent families vs. single people	1.244	1.352
Couples without children vs. single people	1.139	1.105
Couples with children vs. single people	0.961	1.048
Middle vs. higher socio-economic groups	0.925	0.938
Lower vs. higher socio-economic groups	1.160*	1.215**
Overpopulation vs. no overpopulation	1.027	1.081
Work continuity vs. economically inactive	3.772***	4.405***
Telework vs. economically inactive	1.683	1.973
Work stoppage vs. economically inactive	1.238***	1.412***
25–49 vs. 18–25	—	0.940
50–64 vs. 18–25	—	1.057
Over 65 vs. 18–25	—	1.374***
Small and medium-sized towns vs. rural areas	—	1.316
Cities vs. rural areas	—	1.267

*p < .05; **p < .01; ***p < .001.

*Coverage:* all households (*n* = 1967).

These findings are supported by qualitative data providing information about the organisation and reasons for leaving the home by members of the same household. Interview data showed that men more often than women continued to leave the home for personal leisure: for example, hunting for the partner of a female farmer, or jogging for an airline pilot living in the city. Sometimes they left the home to complete tasks that they did not usually carry out before the pandemic, such as shopping for food. Their outings sometimes infringed the new rules, for example by exceeding the authorised distance from the home. They generally described these activities as necessary for personal well-being, or sometimes, among older couples, as a way of protecting the health of a female partner who was self-isolating. The leisure practices of men were also more likely to be justified as part of a daily or weekly schedule, serving as a refuge, whereas the personal leisure activities of women were organised during any time that remained after they had carried out their work-related, domestic and parenting tasks.

In addition to the data provided by the statistical survey, the interview narratives also captured the experience of mothers living through lockdown. Although they mainly regretted the lack of leisure and personal time, they rarely disproved of their partner’s activities outside the home. Moreover, if they went out for a reason other than work, women did not go as far as their partner. They most often stayed at home, including for rare leisure activities, such as reading or watching television, or they remained in the vicinity of the home (courtyard, garden), whatever their social milieu. This was the case for Rosa, a checkout assistant on short-time work and mother of three children, who said she shut herself up in her home with her children while her partner went out to work. Stéphanie, an unemployed administrative worker living with her husband and her 18-year-old daughter, reported that she suffered from boredom while her husband could go out almost every day for professional reasons and shopping activities. Agnès, a mother of four children, whose husband had a higher-level occupation, said that she went no further than the outside of her apartment block or remained in the neighbourhood to help her aunt. In a wealthy couple confined to the countryside during lockdown, recreational outings were organised to visit a neighbour or to have a drink with friends, generally in the presence of the male partner. This was the case for Delphine, an assistant producer who had stop working and had more time available than Christophe, a human resources manager, who was then teleworking.

### Working From Home: Gendered Access to Personal Space

The home became not only a place for leisure pursuits and family life, but also a place for paid work activities. While telework was not a widespread practice in France before the pandemics, it spread considerably during the first lockdown, but in a very unequal way according to the type of work and the level of education (OECD, 2021). The analysis of the COCONEL survey data also shows that conditions for teleworking are highly gendered.

COCONEL was one of few surveys in France to provide information about the conditions of teleworking at home during the pandemic. By May 2020, 29% of the population in employment before lockdown worked from home. This was the case for 86% of those in higher-level occupations. Telework was presented in the public debate as an advantage for well-qualified workers (Leclerc, 2020). But it was also a source of gender inequalities. As already noted, women stopped working more often than men during the first lockdown, regardless of the reason for doing so. Where women continued to work, they did so from home as much as men. The COCONEL survey found that 39% of women working from home shared their workspace with other household members, compared to 24% of men. The gap widened when children were present, with 47% of teleworking mothers sharing their workspace compared to 20% of fathers. By contrast, 45% of fathers teleworked from a room specifically designated as their work space, compared to only 27% of mothers. This result would seem to reflect structural inequalities in employment and pay, especially in higher-level occupations (Georges-Kot, 2020).

In the interviews with respondents in higher and intermediate level occupations, men were found to have appropriated certain rooms in the home, for example a bedroom or study for their work, and sometimes for their leisure. This situation was observed, as anticipated, both among hypergamous couples, where the women did not work or had stopped working, and among homogamous couples, where the women worked in an occupation of a level similar to that of their partner, which was more unusual, for example, in the case of a couple who were both teachers. The interviews showed that the re-distribution of domestic space, whether for work or leisure, often occurred informally, without prior negotiations, thereby revealing the internalisation of male precedence in the use of space:

I had to prepare for my job interview, but I had the opportunity to do so because my husband was at work. So I was able to prepare in the living room, comfortably installed at my desk (Stéphanie, in a couple, one child).
He comes to see us at lunchtime; he pretty much has lunch with us, 20 min. My husband is mainly in his room and in general doesn’t come out. I see him at 1 pm and then towards 9 pm (Agnès, in a couple, four children).
He’s started making sculptures, carving wood, so he spends almost all his time on the patio, morning to evening. Sometimes I tell him: “I need you here, cut that stuff out!” (Jeanne, in a couple, two children).

Some women were able to throw off the shackles of conjugal pressure by choosing not to live with their partner during lockdown, appropriating the entire domestic space for themselves and some of their children. But this option was exceptional, a result both of real-estate ownership and a shared understanding of equality within couples. Overall, these indicators show that mothers have a specific relationship to the home environment, which can be qualified as “domestic imprisonment”. Lockdown, therefore, had a profound impact on living conditions for mothers, with negative consequences for their well-being.

### Well-Being: Gendered Access to Personal Leisure Time and Activities

The COCONEL survey addressed the subjective experience of lockdown for the well-being of working-age mothers. Respondents were questioned about the difficulties they experienced in everyday life. As shown in Table 4, a regression model confirmed that women in couples were more likely than men to experience difficult moments in the day, after controlling for the effects of family structure, social milieu, working arrangement, and housing conditions. Whereas women and men in couples without children said they experienced few difficulties, and differences in gender perceptions were smaller, 62.2% of mothers said they experienced a difficult period during the day, compared to 55.3% of fathers.

**TABLE 4:** Logit: experiencing a difficult period during the day.

Variables	Odds ratios
Women vs. men	1.181*
Single people vs. couples without children	1.581**
Single-parent families vs. couples without children	1.049
Couples with children vs. couples without children	1.646***
Middle vs. higher socio-economic groups	1.223
Lower vs. higher socio-economic groups	1.157
Overpopulation vs. no overpopulation	1.600***
Telework vs. work continuity	1.707
Work stoppage vs. work continuity	1.976***
Economically inactive vs. work continuity	1.419

*p < .05; **p < .01; ***p < .001.

*Coverage:* all (*n* = 1966).

The interviews showed that women felt more vulnerable for two main reasons. Firstly, because they were the principal caregivers for young children during the day, they felt overwhelmed by the situation. Secondly, during lockdown, female respondents who were teleworking or economically inactive found that their partners who continued working contributed little to housework and remote schooling. This was true for Agnès (in a couple, four children), who handled almost all the housework while her partner shut himself up in the bedroom to work. It was also the case for Jeanne (in a couple, two children), who finished her teaching work late at night after spending the day looking after her two young children. She explained: “We don’t have the same daily lives”. Her husband, also a teacher, reported not feeling fatigued at the time of the interview. The second reason for women on short-time work or unemployed with older children lies in their sense of domestic boredom. For example, Rosa (in a couple, three children), a checkout assistant, who was carrying out some household tasks to pass the time, said: “I try something new every day. My children are older and can take care of themselves.” The men interviewed reported fewer cases of personal difficulties, because they were less involved in the additional housework and parenting tasks during lockdown, while also being constrained by gender norms from expressing emotions that might undermine their virile image (Connell and Messerschmidt, 2015).

Lockdown resulted in an increased feeling of social isolation, captured by the question in the COCONEL survey: “Do you currently (during lockdown) feel isolated in your neighbourhood or home?” Table 5 presents the odds ratios for the new feeling of isolation taking into account the working arrangement, social milieu, housing conditions, the presence of children, and outings. On average, slightly more women than men felt isolated, but more fathers than mothers said they were experiencing a new feeling of isolation: 28% of fathers compared to 21.5% of mothers.

**TABLE 5:** Logit: new feeling of isolation.

Variables	Odds ratios
Men with children vs. men without children	1.632*
Women with children vs. women without children	1.511
Women without children vs. men without children	1.042
Middle vs. higher socio-economic groups	1.282
Lower vs. higher socio-economic groups	1.245
Overpopulation vs. no overpopulation	1.630***
Telework vs. work continuity	1.117
Work stoppage vs. work continuity	1.396
Economically inactive vs. work continuity	1.195
18–25 vs. over 65	0.994
25–49 vs. over 65	0.861
50–64 vs. over 65	0.886
Small and medium-sized towns vs. rural areas	1.096
Cities vs. rural areas	1.407***

*p < .05; **p < .01; ***p < .001.

*Coverage:* all individuals in a couple (*n* = 1,233).

Women were less likely than men to feel isolated during lockdown because they were living closer to family members and were able to provide mutual support. The COCONEL survey showed that 38% of women in couples with children had a relative living within 1 km from their home, the authorised geographical limit for outings, compared to 27% for men in couples with children. This percentage was even higher for women with low socio-economic status: 48%, compared to 27% for women with high socio-economic status, confirming that the family played a key protective role among the lower socio-economic group.

The qualitative study underscored the importance of women’s residential preferences in the organisation of daily life among families in lower socio-economic categories. This was the case for Marie-Claire, an employee and remarried, whose house had been rebuilt in part by her father, and for Séverine, 55, a farmer in Burgundy, who had inherited the family farm. Women were also more involved in maintaining local relationships and sociability (Authier and Cayouette-Remblière, 2021). The fact that social life was restricted during lockdown to a local neighbourhood meant that women in this situation were less affected by new feeling of isolation.

For men from all social class, lockdown caused a greater disruption in their lifestyle since the increased amount of time spent with children did not compensate for the lack of sociability outside the family. The combined findings from the survey and interviews confirmed that male sociability was more likely to be structured by professional life and work colleagues, whereas women more often maintained contacts with their relatives. Women were also more likely to receive friends and relatives in their homes or meet at private or semi-private venues.

## Discussion and Conclusions

One of the aims of the study was to explore whether the pandemic might have increased gender inequalities in France due to its impact on working arrangements and living conditions, including teleworking, access to personal space, leisure time and activities, and to family support networks. In seeking to achieve this aim, the researchers adopted two working hypotheses to frame their analysis. Firstly, they asked whether, despite substantial public support in France to avoid mass unemployment and the exit of working parents from the labour market, working-age women with children had been more adversely affected by lockdown measures than men in the same situation across socio-economic categories. Secondly, they analysed the impact of lockdown on dimensions of social life other than employment, and asked how lockdown had affected the quality of life and well-being of working mothers.

COCONEL is among the few sociological surveys to be conducted using a random sample of national population that simultaneously takes into account working arrangements, living conditions and well-being, and allows for an intersectional analysis of social inequalities by gender and socio-economic status. The results presented in this article are based on the sixth wave of the survey documenting employment, living conditions and well-being during the first wave of the pandemic and lockdown. The findings showed that working-age mothers were more likely than their male counterparts to stop working during lockdown. When controlled by age, socio-economic category and place of residence, belonging to the lower socio-economic groups was found to be associated with a higher risk of stopping work during lockdown. Highly educated women were less affected by firm closures than women with a low level of education, since they were more often able to work from home. These results are in line with findings from other literature about the gendered impact of COVID-19 on the labour market (Adams-Prassl et al., 2020).

Data on living conditions and social well-being were included in the analysis as potential factors exacerbating gender inequalities. The authors measured the gendered access to personal spaces for women and men working from home, gendered access to personal leisure time and activities, and the legitimacy of this differential access, and gendered and class-based access to family support networks during lockdown. The study found that men left the home more frequently during the day than women during lockdown, and that these differences increased when children were present. When controlled for employment, household composition, age and socio-economic status, men were found to be 1.6 times more likely to go out during the day than women. The same pattern was observed for well-being. Working age mothers more often than their male counterparts reported difficulties during the day, but they less frequently felt isolated. These findings suggest that mothers’ difficulties during confinement were more likely to be related to the additional domestic and parental work at home and less to psychological distress, while fathers’ new difficulties were related to the disruption of social contacts with family and others.

The design of the COCONEL survey did not allow a textured analysis of all the variables of interest to the researchers. Like all national population-based surveys, the data failed to capture the experience of highly vulnerable groups such as lone mothers who were particularly affected by the pandemic. In addition, the analyses were based on cross-sectional data limited to the period April−May 2020 rather than longitudinal data. Nor did COCONEL enable comparisons to be made of the situation between men and women within couples. The authors used the findings from in-depth interviews carried out during the first lockdown to address some of these limitations and better understand the mechanisms that contribute to the deterioration of the situation of working-age mothers. They were able to use a panel of respondents who were already being followed prior to the pandemic to complement the quantitative analyses by providing biographical and longitudinal data.

The respondents’ narratives suggest two main mechanisms – socio-economic and employment status − that contributed to the deterioration of the situation of working-age mothers. Among the higher socio-economic groups, with their more spacious dwellings, women mainly kept their jobs and worked from home, generating a dual domestic−occupational workload that eroded their well-being, given that the customary outsourcing of domestic work was no longer possible. They tended to feel overwhelmed by the lack of time for themselves or their leisure activities. However, they did not reproach their spouses for appropriating the domestic space and for their limited contribution to domestic work. It seemed that the crisis delegitimised any expression of female protest.

In the lower socio-economic groups, where housing conditions are less amenable to homeworking, more women stopped working or were put on reduced hours. Despite the loss of income, the impact on the deterioration of individual and family well-being appeared to be mitigated by their closer family networks and their lesser reliance on paid housework and childcare services before the pandemic. Women with a lower level of education complained less about additional domestic and parenting tasks and more about being bored at home after being required to stop working for several weeks. They were eager to return to work for the benefits of socialisation and social identity that it conveyed.

The findings from the study shed new light on the dynamics of gender inequality and its underlying mechanisms during the COVID-19 pandemic. While most of the available economic literature emphasises the role of sex segregation on the labour market and public policies, this study highlights the importance of gender norms and their impact on the appropriation of space and family resources by men in the private sphere (Bessiere and Gollac, 2020). It also reveals the role of access to personal support networks during lockdown, which was found to vary across socio-economic groups and gender, and served to mitigate the impact on individual well-being.

By calling into question the gender inequalities that occurred in the private sphere, and not only employment outcomes, these findings about changes in gender inequalities during the early stages of the pandemic have broader policy implications beyond the current pandemic. They suggest the importance of reconsidering and value of the major role of women in creating and maintaining close ties with the family and in the neighbourhood, and more broadly their role in providing emotional support to the family during the health crisis. It also suggests the need for enterprises to strengthen professional equality policies that fully recognise parenting time and the support for dependants. They touch on how society supports women’s autonomy and well-being in the longer term, through childcare facilities and work−life balance policies, and are, therefore, important in informing future public policy development.

## Data Availability

Anonymised data from the analysis of the sixth wave of the COCONEL survey can be made available by the corresponding author on request.
